# Evaluation of BNT162b2 Vaccine Effectiveness in Galicia, Northwest Spain

**DOI:** 10.3390/ijerph19074039

**Published:** 2022-03-29

**Authors:** Jacobo Pardo-Seco, Narmeen Mallah, Luis Ricardo López-Pérez, Juan Manuel González-Pérez, Benigno Rosón, María Teresa Otero-Barrós, Carmen Durán-Parrondo, Carmen Rodríguez-Tenreiro, Irene Rivero-Calle, Alberto Gómez-Carballa, Antonio Salas, Federico Martinón-Torres

**Affiliations:** 1 Genetics, Vaccines and Pediatric Infectious Diseases Research Group (GENVIP), Instituto de Investigación Sanitaria de Santiago and Universidad de Santiago de Compostela (USC), 15706 Santiago de Compostela, Spain; j.pardoseco@gmail.com (J.P.-S.); narmeen.mallah@sergas.es (N.M.); carmentenreiro@hotmail.es (C.R.-T.); irene.rivero.calle@sergas.es (I.R.-C.); alberto.gomez.carballa@sergas.es (A.G.-C.); antonio.salas@usc.es (A.S.); 2 WHO Collaborating Centre for Vaccine Safety, 15706 Santiago de Compostela, Spain; 3 Centro de Investigación Biomédica en Red de Enfermedades Respiratorias (CIBERES), 28089 Madrid, Spain; 4 Subdirección de Sistemas y Tecnologías de la Información, Servizo Galego de Saúde, 15703 Santiago de Compostela, Spain; luis.ricardo.lopez.perez@sergas.es (L.R.L.-P.); juan.manuel.gonzalez.perez@sergas.es (J.M.G.-P.); benigno.roson.calvo@sergas.es (B.R.); 5 Dirección Xeral de Saúde Pública, Consellería de Sanidade, 15703 Santiago de Compostela, Spain; maria.teresa.otero.barros@sergas.es (M.T.O.-B.); carmen.duran.parrondo@sergas.es (C.D.-P.); 6 Translational Pediatrics and Infectious Diseases, Hospital Clínico Universitario and Universidad de Santiago de Compostela (USC), 15706 Santiago de Compostela, Spain; 7 Unidade de Xenética, Instituto de Ciencias Forenses (INCIFOR), Facultade de Medicina, Universidade de Santiago de Compostela, 15782 Santiago de Compostela, Spain; 8 GenPoB Research Group, Instituto de Investigaciones Sanitarias, Hospital Clínico Universitario de Santiago (SERGAS), 15706 Santiago de Compostela, Spain

**Keywords:** SARS-CoV-2, COVID-19, vaccine effectiveness, population study

## Abstract

Investigating vaccine effectiveness (VE) in real-world conditions is crucial, especially its variation across different settings and populations. We undertook a test-negative control study in Galicia (Northwest Spain) to assess BNT162b2 effectiveness against acute respiratory syndrome coronavirus 2 (SARS-CoV-2) infection as well as COVID-19 associated hospitalization, intensive care unit (ICU) admission and mortality. A total of 44,401 positive and 817,025 negative SARS-CoV-2 test results belonging to adults were included. Adjusted odds ratios of vaccination and their 95% confidence interval (CI) were estimated using multivariate logistic-regression models. BNT162b2 showed high effectiveness in reducing SARS-CoV-2 infections in all age categories, reaching maximum VE ≥ 14 days after administering the second dose [18–64 years: VE = 92.9% (95%CI: 90.2–95.1); 65–79 years: VE = 85.8% (95%CI: 77.3–91.9), and ≥80 years: VE = 91.4% (95%CI: 87.9–94.1)]. BNT162b2 also demonstrated effectiveness in preventing COVID-19 hospitalization for all age categories, with VE more pronounced for those aged ≥80 years [VE = 60.0% (95%CI: 49.4–68.3)]. Moreover, there was a considerable reduction in ICU admission [VE = 88.0% (95%CI: 74.6–95.8)] and mortality [VE = 38.0% (95%CI: 15.9–55.4)] in the overall population. BNT162b2 showed substantial protection against SARS-CoV-2 infections and COVID-19 severity. Our findings would prove useful for systematic reviews and meta-analysis on COVID-19 VE.

## 1. Introduction

In Spain, the vaccination program against severe acute respiratory syndrome coronavirus 2 (SARS-CoV-2) was launched on 27 December 2020 right after the approval of BNT162b2 vaccine by the European Medicines Agency (EMA) on 21 December 2020 [1]. As of 18 March 2021, three vaccines have been approved by EMA: BNT162b2 (21 December 2020) mRNA-1273 (6 January 2021) and ChAdOx1 (29 January 2021). BNT162b2 and mRNA-1273 contain a messenger RNA (mRNA) molecule that encodes a protein from SARS-CoV-2, while ChAdOx1 contains the SARS-CoV-2 spike protein gene, which instructs the host cells to produce the protein of the S-antigen unique to SARS-CoV-2 [1]. A full vaccine course of BNT162b2, mRNA-1273, or ChAdOx1 nCoV-19 consists of two injections with a predefined time separation between the first and the second dose.

When COVID-19 vaccine supplies were limited, vaccination in Spain against COVID-19 followed a priority scale to reduce the case fatality rate as well as the burden on essential services. The highest priority was given to staff and residents of nursing homes (vaccinated with BNT162b2), followed by: (i) frontline healthcare workers (vaccinated with BNT162b2 or mRNA-1273); (ii) elderly individuals (vaccinated with BNT162b2); (iii) non-essential healthcare workers, education staff, security law corps (vaccinated with ChAdOx1); and finally (iv) other groups by decreasing age [2].

According to the established priority order and the vaccine type assigned to each age group, the vast majority of the Spanish vaccinated population until 18 March 2021 had received one or two doses of BNT162b2. Though clinical trials had been already carried out to measure the short-term efficacy of this vaccine prior to its approval [3], observational studies are required to determine vaccine effectiveness (VE) in diverse populations and real-world settings [4]. Several studies from different countries evaluated BNT162b2 effectiveness, and a related meta-analysis reported a substantial protective effect of BNT162b2 against COVID-19 with VE of 53% ≥ 14 days following the first dose and 95%  ≥ 7 days after the second dose [5].

It has been shown that variations in adherence to vaccine doses and time intervals for dose administration, as well as differences in access to healthcare, SARS-CoV-2 testing, hospitalization threshold and COVID-19 management across countries and settings may influence the external validity of reported VE [6]. An evaluation of VE in different settings and population would therefore prove useful for future systematic reviews and meta-analysis.

Spain is divided into 17 autonomous communities and two autonomous cities, with diverse sociodemographic characteristics. Though the same vaccines have been administered in all Spanish territory, the epidemiological situation with respect to the COVID-19 pandemic and the applied nonpharmaceutical measures have widely varied across the different Spanish regions.

In the present study, we aim to evaluate, for the first time, BNT162b2 VE in Galicia (Northwest Spain) through a test-negative case-control design. We examined BNT162b2 VE against SARS-CoV-2 infection, and COVID-19 severity in a population consisting of 2,280,288 vaccinated and unvaccinated adults. We also undertook stratified analyses by age, number of administered vaccine doses and time since vaccine administration.

## 2. Materials and Methods

### 2.1. Study Design

We carried out a retrospective test-negative control study in Galicia, Northwest Spain [7] to study BNT162b2 VE related to SARS-CoV-2 infection. Test-negative case-control designs are widely used to estimate VE against respiratory viruses [8].

### 2.2. Data Collection

The study encompassed all adults (≥18 years old) who were vaccinated between 27 December 2020 and 18 March 2021. The vaccination program was introduced in Galicia on 27 December 2020, prioritizing first-line health personnel, elderly people (80 years or older) and residents of care homes [9]. COVID-19 vaccination data is available in the Galician public health system (SERGAS). Data were extracted from SERGAS using randomly assigned pseudo-anonymized codes. The authors were blind to the code assignment. Extracted data included age, gender, SARS-CoV-2 test history (date, type of test and result), history of patients’ hospitalization, admission to intensive care unit (ICU) or death by SARS-CoV-2 since the start of the study. Vaccination information was also obtained (vaccine name, number of administered doses and date of vaccine administration).

### 2.3. Exposure and Outcome Ascertainment

The start date of the vaccination campaign in Spain, 27 December 2020, was established as an index date. Individuals who had a positive laboratory-confirmed SARS-CoV-2 test result before the index date were excluded from the analysis. Only a positive SARS-CoV-2 test result was considered per individual, provided that the test took place after the index date. When several positive tests existed for the same individual, only the first positive test was considered. The same individual could serve as one or more non-cases depending on the number of corresponding negative tests. For instance, an individual who had three negative tests after the index date could contribute by three non-case observations to the study.

**Figure 1:**
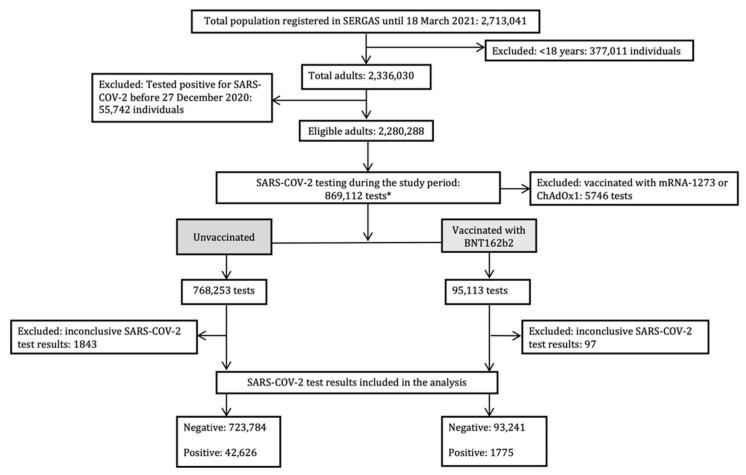
Flow diagram of participants’ selection and SARS-CoV-2 test inclusion in the study. 27 December 2020 was established as the index date for case and non-case selection. The study took place between 27 December 2020 and 18 March 2021. *: the same individual could contribute with more than one test to the study.

The vaccination status was assigned depending on the number of vaccine doses administrated before test date. As BNT162b2 was the first administered vaccine in Spain, most of the participants had received this vaccine during the study period. Therefore, the number of individuals who had received mRNA-1273 and ChAdOx1 vaccines and subsequently underwent SARS-CoV-2 test during the study period was not sufficient to be considered in the analysis (Figure 1). The vaccination status (unvaccinated, partially vaccinated, or fully vaccinated) was assigned according to the vaccination status of the individual on the test date. Subjects who did not receive any dose of the vaccine before the test date were classified as “unvaccinated”. Subjects who had received only one vaccine dose before the test date were considered “partially vaccinated”. Individuals who received the two doses of the vaccine before the test date were deemed “fully vaccinated”.

### 2.4. Statistical Analysis

VE was measured by comparing the odds of vaccination between cases and non-cases. Adjusted odds ratios (ORs) of testing positive for SARS-CoV-2 among vaccinated patients was compared to that of unvaccinated patients using a logistic regression model. The time since vaccine administration was introduced in the model as an independent variable. This time was represented by zero days for unvaccinated individuals, and the following vaccination periods were considered for vaccinated individuals: 1–6 days after the first dose, 7–13 days after the first dose, 14–20 days after the first dose, 1–6 days after the second dose, 7–13 days after the second dose, and 14 days or more after the second dose. The models were adjusted for the date of SARS-CoV-2 test (in weekly units since the start of study: December 27th, 2020), age and sex. VE was then calculated and expressed in percentages as follows: VE = (1 − OR) × 100.

To evaluate COVID-19 severity in the vaccinated population, we compared the odds of hospitalization, ICU admission and hospital mortality between SARS-CoV-2 positive-tested individuals who received BNT162b2 to those who tested positive but who had not been vaccinated. Patients with nosocomial infections were excluded from this analysis, since SARS-CoV-2 infection could not be ascertained as the cause of hospitalization. Again, adjusted ORs and their 95% CI were computed using logistic-regression models. The models were adjusted for age, date of positive SARS-CoV-2 test and sex. Upon stratifying the participants according to vaccination status and outcome, only those groups with at least five observations in each arm were considered.

The analyses were stratified by age group (18–64, 65–79 and ≥80 years) when it was possible.

All analyses were carried out using the statistical software R (v. 4.0.3) (University of Auckland, Auckland, New Zealand) [10].

## 3. Results

### 3.1. Study Population

At the start of the study (27 December 2020), the total number of participants recorded in SERGAS was 2,713,041, out of whom 2,336,030 were aged 18 years and above. A total of 55,742 individuals had a positive laboratory-confirmed SARS-CoV-2 test result before the index date and were excluded from the analysis. This decision was taking to account the variation in criteria applied for individuals’ vaccination throughout the vaccination campaign, and was in line with procedures carried out in previous studies [11,12]. A total of 2,280,288 individuals were assessed for eligibility during the study period (27 December 2020–18 March 2021). From these, 250,226 received at least one of the approved vaccines. Most of those individuals (*n* = 169,104; 67.6%) were recipients of BNT162b2 (two doses: *n* = 95,890; one dose: *n* = 73,214), almost one-third (*n* = 73,658; 29.4%) received the first dose of ChAdOx1 and only 3.0% (*n* = 7464) were vaccinated with mRNA-1273 (two doses: *n* = 3729; one dose: *n* = 3735). Therefore, only individuals vaccinated with BNT162b2 were included in the analysis.

A total of 596,850 SARS-CoV-2 test results (44,401 positive and 552,449 negative) fulfilled the inclusion criteria (Figure 1). A total of 78.5% of SARS-CoV-2 tests were performed in the population aged between 18 and 64 years; 12.0% corresponded to individuals in the age range of 65–79; and 9.5% belonged to individuals ≥80 (Table 1). More than half of the eligible test results (57.7%) belonged to women (Table 1).

### 3.2. BNT162b2 Vaccine Effectiveness against SARS-CoV-2 Infection

**Figure 2:**
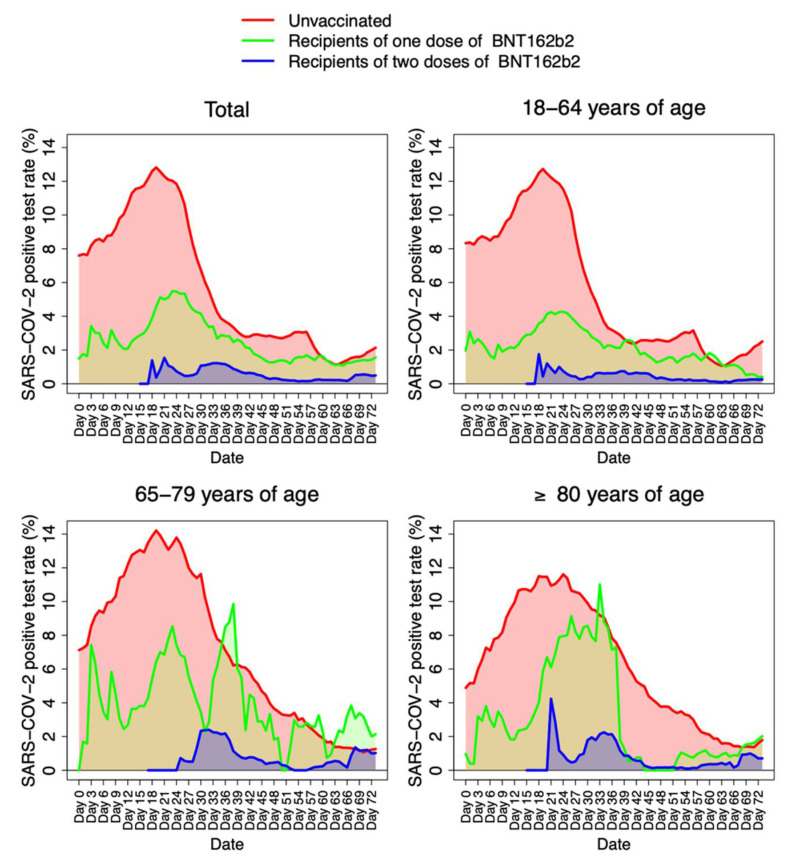
Frequency of weekly positive SARS-CoV-2 tests computed by age groups, and number of administered BNT162b2 vaccine doses. The red line represents the unvaccinated population. The green line represents those individuals who received one dose of BNT162b2. The blue line represents those individuals who received two doses of BNT162b2.

As shown in Figure 2, SARS-CoV-2 positivity rate among the vaccinated population declined with time. A substantial decrease in the odds of SARS-CoV-2 infection was observed in all age categories since the first week (1–6 days) after receiving the first dose of BNT162b2 (Table 2).

The greatest VE against SARS-CoV-2 infection was observed ≥14 days after receiving the second dose of BNT162b2 [VE = 91.0% (95% CI: 89.0–93.0)] (Table 2). Stratifying this result by age, revealed that the VE is substantial for all age categories: VE = 93.0% (95% CI: 90.0–95.0) for 18–64 years; VE = 85.0% (95% CI: 75.0–91.0) for 65–79 years; and VE = 91.0% (95% CI: 87.0–94.0) for ≥80 years (Table 2).

### 3.3. BNT162b2 Vaccine Effectiveness against COVID-19 Severity

Among 44,401 SARS-CoV-2 positive-tested individuals (Table 1), 606 were excluded due to nosocomial infections.

**Figure 3:**
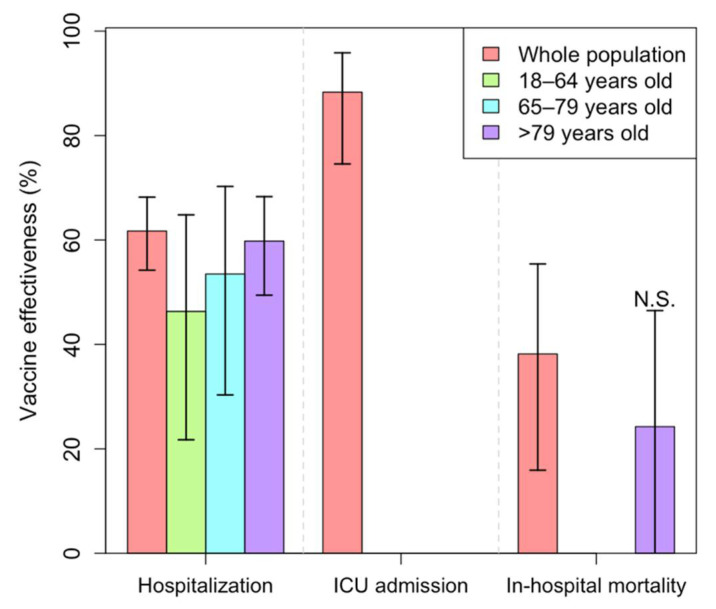
Vaccine’s effectiveness results ogBNT162b2 vaccine. 95% CI are displayed in black lines. Extended results in Table S2. N.S.: not significant.

COVID-19 was deemed as the cause of hospitalization in 4254 of the remaining 43,795 adults. A total of 4103 of the 4254 hospitalized adults were unvaccinated and the remaining 151 were vaccinated with BNT162b2 (one dose: 127; two doses: 24) (Table S1). A considerable reduction in hospital admissions for COVID-19 was detected in individuals vaccinated with any dose (one or two) of BNT162b2 as compared to unvaccinated individuals [VE = 62.0% (95% CI: 54.2–68.2)] (Figure 3, Table S2). BNT162b2 administration showed protection against COVID-19 hospitalization for all age categories, with the VE more pronounced for those aged 80 years and older [18–64 years: VE = 46.0% (95% CI: 21.7–64.8); 65–79 years: VE = 53.0% (95% CI: 30.3–70.3), and ≥80 years: VE = 60.0% (95% CI: 49.4–68.3)] (Figure 3, Table S2).

Due to the limited number of positive-tested individuals who had received two doses of BNT162b2 during the study period, we could not stratify the COVID-19 severity analysis by the number of received doses.

Among 1762 BNT162b2 vaccinated and SARS-CoV-2 positive individuals, only five incompletely vaccinated (one dose) adults younger than 80 years old were admitted to ICU (Figure 3, Table S2). For this reason, BNT162b2 VE against ICU admission could not be stratified by age, but globally, the vaccine showed high effectiveness against ICU admission for COVID-19 [VE = 88.0% (95% CI: 74.6–95.8)] (Figure 3, Table S2).

Overall, the BNT162b2 vaccine was associated with a decrease in death for COVID-19 [VE = 38.0% (95% CI: 15.9–55.4)] (Figure 3, Table S2). Due to the limited number of registered COVID-19 deaths, the analysis could be only stratified for the age group ≥ 80 years. The BNT162b2 vaccine was suggested to be associated with 24% lower odds of COVID-19 mortality in vaccinated individuals ≥80 years as compared to unvaccinated individuals from the same age category, yet the confidence interval was large, probably due to the insufficient number of observations [OR = 0.76 (95% CI: 0.54–1.05)] (Figure 3, Table S2).

## 4. Discussion

We provided early real-world evidence of BNT162b2 effectiveness in reducing SARS-CoV-2 infections as well as COVID-19 disease severity in the general adult population in Galicia, Spain. Our findings reveal a substantial protection against SARS-CoV-2 infections in all age categories, a few days after receiving the first dose (one to six days). BNT162b2 VE against SARS-CoV-2 reached its peak ≥14 days after the second dose; exceeding 90% in the elderly population (≥80 years) and in those aged 18–64 years. Remarkable oscillations of SARS-CoV-2 positive tests were observed for partially vaccinated individuals of the age groups 65–79 and ≥80 years. These findings can be related to COVID-19 outbreak events at nursing homes at the start of the vaccination campaign. These events mainly involved residents, being most of them asymptomatic (according to media news). However, it is also remarkable that these outbreaks did not affect fully vaccinated individuals.

A reduction in SARS-CoV-2 infections implies a decrease in hospital and ICU admissions, and consequently a protection against death caused by COVID-19. This is in line with our observation that the odds of hospital admission for COVID-19 are 62% lower in individuals vaccinated with one or two doses of BNT162b2 than in nonvaccinated individuals. Our findings also point to overall reduced odds of ICU admissions and mortality for COVID-19. Nonetheless, upon categorizing the study participants who were admitted to ICU or died from COVID-19 according to their age, a limited number of observations were left in each age category, restraining therefore the stratification of this analysis by age. Replicating the study in a larger population would allow for a comprehensive evaluation of BNT162b2 effectiveness against ICU admissions and mortality caused by COVID-19.

Our findings coincide with those of previous studies that measured BNT162b2 VE. In the United Kingdom, Pritchard et al. demonstrated 80% VE of BNT162b2 against SARS-CoV-2 infections among individuals fully vaccinated as compared to non-vaccinees [13]. Another study in Israel involving healthcare workers, reported a 75% reduction in SARS-CoV-2 infections in the 15 to 28 days after receiving the first dose of BNT162b2 [12]. In England, SARS-CoV-2 infections declined by 89% in elder adults after ≥14 days of vaccinating with the second dose of BNT162b2 [14]. In Scotland, the hospitalization rate for COVID-19 also decreased by 91% in the 28 to 34 days after the administration of the first dose of BNT162b2 [11]. In a study undertaken in nursing homes, Shrotri et al. estimated a 65% decrease in SARS-CoV-2 infections among residents of English long-term care facilities, 35 to 48 days after receiving the first doses of BNT162b2 [15].

In Spain, since the start of COVID-19 vaccination program on 27 December 2020, some studies have evaluated VE. Nonetheless, the studies targeted various populations and/or socioeconomically different regions where the COVID-19 epidemiological situation and the related preventive measures were dissimilar. Cabezas et al. evaluated SARS-CoV-2 infections in a group of residents and healthcare workers of nursing homes in Catalonia (Spain) until 5 March 2021 and reported a 91% and 80% reduction in the infection rate after receiving two doses of BNT162b2 for nursing home residents and staff, respectively [16]. Two other studies in the elderly population were conducted in Spain using national vaccination registers [17,18]. Both studies also provided an early evaluation of VE and were concluded by 10 March 2021 [17] and 4 April 2021 [18]. The study of Mazagatos et al. showed high effectiveness of mRNA vaccines in preventing COVID-19 hospitalizations (88%) and deaths (97%) [18]. Likewise, Monge et al. showed substantial VE against SARS-CoV-2 infections in individuals with and without previous infection (57% and 82%, respectively) [17]. Almost all administered vaccines (99.8%) in that study were BNT162b2 [17]. In Navarre (Spain), Martínez-Baz et al. estimated VE in a population of adults who were in close contact with COVID-19 positive cases between January and April 2021. The study excluded residents of nursing homes and the majority of the participants had received BNT162b2 vaccine [19]. Martínez-Baz et al. showed that full vaccination reduces SARS-CoV-2 infections by 66%, symptomatic COVID-19 by 42% and hospitalization for COVID-19 by 72% [19].

Our findings are unlikely to have been biased. First, since our study is population-based, selection bias is unlikely to have occurred as we had access to the whole population registered in SERGAS. In Galicia, vaccination took place through SERGAS and was made freely available to everyone. All data were electronically registered through sanitary cards that are unique to each person registered in SERGAS. Therefore, exposure misclassification is also a remote possibility. As for outcome ascertainment, misclassification is also improbable, the infection with SARS-CoV-2 and the disease severity were determined through laboratory-based tests and related medical records. Furthermore, confounding bias is unlikely to have occurred, as we adjusted the estimated VE for potentially confounding factors including sex, age and duration of pandemic.

Our study suffers from the following limitations. We did not have data about patients’ comorbidities and COVID-19 symptoms (symptomatic/asymptomatic, and time lapse between symptoms’ appearances and COVID-19 testing). In addition, different nonpharmaceutical measures were applied by the government during the study period, which might have influenced the incidence of SARS-CoV-2 infections. During the COVID-19 pandemic, the Spanish government implemented various nonpharmaceutical interventions based on a four-level pandemic-alert scale that uses the cumulative incidence level proposed by the World Health Organization (WHO) and by the European Center for Disease Prevention and Control (ECDC). The measures included, among others: total lockdown, border closure of autonomous communities, provinces or municipalities, personal protective measures, night-time curfew, mass-gathering cancellations, control of numbers of individuals (capacity limitations) and limited opening hours in specific facilities such as restaurants. Our study also lacks data about the identity of detected SARS-CoV-2 variants in the positively tested individuals [20,21,22]. Individuals in different settings could be exposed at a greater or lesser extent to SARS-CoV-2, hence stratifying the analysis according to patients’ settings such as homecare centers, frontline healthcare workers and administrative occupation would provide a deeper insight on VE. We did not have information on SARS-CoV-2 viral load. SARS-CoV-2 virus quantitation can help understand disease heterogeneity and aid in identifying patients who may benefit from new COVID-19 therapies [23]. Although our study was population-based and encompassed a large sample size, the number of positive SARS-CoV-2 tests was not sufficient to stratify the analysis of VE against ICU admission and mortality for COVID-19 by different age subgroups, hence replicating the study in a larger population would allow to comprehensively measure VE against severe COVID-19 outcomes. Our findings cannot be generalized to disadvantaged populations who have limited access to healthcare services, as in Spain access to healthcare is universal. Nonetheless, confounding bias due to healthcare-seeking behavior is unlikely to have occurred in our study [6].

Evaluating BNT162b2 safety was beyond the scope of the present study, yet the authors recommend to investigate the adverse effects related to this vaccine in the Spanish population in future studies. BNT162b2 was previously associated with increased risk of serious adverse events such as myocarditis and lymphadenopathy [24]. Individuals vaccinated with BNT162b2 also reported side effects such as soreness, fatigue, myalgia, headache, fever and muscle pain [25].

## 5. Conclusions

Our study shows that BNT162b2 offers substantial protection against SARS-CoV-2 infection from the moment of vaccination, leading to an important reduction in COVID-19 associated hospitalization, ICU admission and mortality. The findings of the present study would prove useful for systematic reviews and meta-analysis on the topic. However, studies on VE should be routinely updated until the end of the pandemic to provide a more comprehensive analysis on protection against COVID-19 severity, new emerging SARS-CoV-2 variants and duration of acquired immunity. Further studies are also required to study the overall vaccine performance and evaluate vaccine safety in different settings.

## Figures and Tables

**Table 1:** Description of included SARS-CoV-2 tests stratifying by age, sex, vaccination status and test result.

	No. of Participants		Vaccination Status											
			Unvaccinated (*n* = 766,410)				Dose 1 (*n* = 42,999)				Dose 2 (*n* = 52,017)		Dose 2 (*n* = 52,017)	
			Negative Test		Positive Test		Negative Test		Positive Test		Negative Test		Positive Test	
	*n*	%	*n*	%	*n*	%	*n*	%	*n*	%		%	*n*	%
Whole population	861,426	-	723,784	94.4%	42,626	5.6%	41,497	96.5%	1502	3.5%	51,744	99.5%	273	0.5%
Age category (Years)														
18–64	680,312	78.5%	583,526	94.8%	32,285	5.2%	26,499	97.0%	809	3.0%	31,381	99.6%	122	0.4%
65–79	104,198	12.0%	88,491	93.1%	6598	6.9%	3735	95.3%	183	4.7%	5108	99.2%	41	0.8%
≥80	82,650	9.5%	51,767	93.3%	3743	6.7%	11,263	95.7%	510	4.3%	15,255	99.3%	110	0.7%
Sex														
Male	366,578	42.3%	322,178	94.7%	20,116	5.3%	10,220	96.5%	366	3.5%	12,356	99.5%	82	0.5%
Female	500,567	57.7%	401,591	94.1%	22,510	5.9%	31,277	96.5%	1502	3.5%	39,388	99.3%	191	0.7%
Missing	15	0.0%	15	100%	-	-	-	-	-	-	-	-	-	-

**Table 2:** BNT162b2 vaccine effectiveness in preventing SARS-CoV-2 infection stratified by age, time since vaccine administration and number of received doses.

	Whole Population		18–64 Years		65–79 Years		≥80 Years	
Vaccination Status	OR	VE	OR	VE	OR	VE	OR	VE
	(95% CI) ^1^	(95% CI)	(95% CI) ^1^	(95% CI)	(95% CI) ^2^	(95% CI)	(95% CI) ^2^	(95% CI)
Unvaccinated	1	-	1	-	1	-	1	-
1–6 days dose 1	0.3	70.50%	0.37	63.10%	0.2	80.50%	0.19	81.30%
	(0.26–0.33)	(66.5–74.1)	(0.32–0.42)	(57.5–68.1)	(0.12–0.31)	(69–88.6)	(0.13–0.26)	(74.3–86.8)
7–13 days dose 1	0.36	64.30%	0.36	63.80%	0.38	62.90%	0.43	57.90%
	(0.33–0.39)	(61.2–67.2)	(0.32–0.40)	(59.7–67.7)	(0.29–0.47)	(53.2–71.2)	(0.36–0.50)	(50.8–64.1)
14–20 days dose 1	0.32	67.70%	0.25	74.70%	0.43	57.40%	0.5	51.40%
	(0.29–0.35)	(64.6–70.6)	(0.22–0.29)	(71.0–78.0)	(0.34–0.55)	(46.1–66.9)	(0.42–0.58)	(43.5–58.4)
1–6 days dose 2	0.18	81.60%	0.17	83.40%	0.22	78.70%	0.16	84.70%
	(0.15–0.22)	(77.7–85.0)	(0.12–0.22)	(78.3–87.6)	(0.12–0.36)	(65.1–88.1)	(0.11–0.22)	(78.9–89.3)
7–13 days dose 2	0.25	75.40%	0.21	79.00%	0.17	83%	0.22	78.80%
	(0.20–0.30)	(70.1–80.1)	(0.15–0.28)	(71.7–84.9)	(0.09–0.29)	(71.2–90.9)	(0.16–0.29)	(71.7–84.6)
≥14 days dose 2	0.09	90.80%	0.07	92.90%	0.15	85.80%	0.09	91.40%
	(0.07–0.11)	(88.6–92.7)	(0.05–0.10)	(90.2–95.1)	(0.08–0.23)	(77.3–91.9)	(0.06–0.12)	(87.9–94.1)

CI: confidence interval; OR: Adjusted odds ratio; VE: vaccine effectiveness. ^1^ odds ratio adjusted for sex, age and time period between SARS-CoV-2 test and the start of study (in weeks). ^2^ odds ratio adjusted for sex and time period between SARS-CoV-2 test and the start of study (in weeks).

## Data Availability

Not applicable.

## Supplementary Materials

The following supporting information can be downloaded at: https://www.mdpi.com/article/10.3390/ijerph19074039/s1, Table S1: Distribution of COVID-19 attributed hospitalization, intensive care unit (ICU) admission and mortality among SARS-CoV-2 positive cases stratified by vaccination status, age and sex.; Table S2: BNT162b2 VE against COVID-19 associated hospitalization, intensive care unit (ICU) admission and mortality stratified by age.
