# Supplementary material for: Evaluation of BNT162b2 Vaccine Effectiveness in Galicia, Northwest Spain
Source: Int J Environ Res Public Health. 2022 Mar 29;19(7):4039. doi: 10.3390/ijerph19074039 (PMC8998680; doi:10.3390/ijerph19074039)
Supplement: Supplementary file 1 [file ijerph-19-04039-s001.zip › ijerph-1634424-supplementary.pdf]

Table S1. Distribution of COVID-19 attributed hospitalization, intensive care unit (ICU) admission and mortality among SARS-CoV-2 positive cases stratified by vaccination status, age and sex.

	**COVID-19 hospitalization**						**COVID-19 ICU admission**						**COVID-19 mortality***					
	**Unvaccinated (*n* = 42033)**		**BNT162b2 Dose 1 (*n* = 1491)**		**BNT162b2 Dose 2 (*n* = 271)**		**Unvaccinated (*n* = 42033)**		**BNT162b2 Dose 1 (*n* = 1491)**		**BNT162b2 Dose 2 (*n* = 271)**		**Unvaccinated (*n* = 42033)**		**BNT162b2 Dose 1 (*n* = 1491)**		**BNT162b2 Dose 2 (*n* = 271)**	
	n	%	*n*	%	*n*	%	*n*	%	*n*	%	*n*	%	*n*	%	*n*	%	*n*	%
Whole population	4103	9.8%	127	8.5%	24	8.9%	677	1.6%	5	0.3%	0	0%	513	1.2%	43	2.9%	8	3.0%
Age category (Years)																		
18–64	1645	5.1%	24	3.0%	2	1.6%	337	1.0%	3	0.4%	0	0%	38	0.1%	1	0.1%	1	0.8%
65–79	1316	20.6%	20	10.9%	5	12.2%	318	5.0%	2	1.1%	0	0%	144	2.3%	3	1.6%	1	2.4%
≥80	1142	32.4%	83	16.5%	17	15.7%	22	0.6%	0	0%	0	0%	331	9.4%	39	7.8%	6	5.6%
**Sex**																		
Male	2230	11.3%	47	13.1%	8	9.9%	462	2.3%	2	0%	0	0%	308	1.6%	14	3.9%	2	2.5%
Female	1873	8.4%	80	7.1%	16	8.4%	215	1.0%	3	0%	0	0%	205	0.9%	29	2.6%	6	3.2%

* only hospital-based mortalities were included in the analysis

Table S2: BNT162b2 VE against COVID-19 associated hospitalization, intensive care unit (ICU) admission and mortality stratified by age.

		**COVID-19 hospitalization**						**COVID-19 ICU admission**						**COVID-19 mortality***					
		No		Yes		OR (95% CI)	VE (95% CI)	No		Yes		OR (95% CI)	VE (95% CI)	No		Yes		OR (95% CI)	VE (95% CI)
	Vaccinated	*n*	%	*n*	%			*n*	%	*n*	%			*n*	%	*n*	%		
Whole population	No	37930	90.2%	4103	9.8%	1	-	41356	98.4%	677	1.6%	1	-	41520	98.8%	513	1.2%	1	-
	Yes	1611	91.4%	151	8.6%	0.38 (0.32–0.46)	62.0% (54.2–68.2)	1757	99.7%	5	0.3%	0.12 (0.04–0.25)	88.0% (74.6–95.8)	1711	97.1%	51	2.9%	0.62 0.45–0.84)	38.0 (15.9–55.4)
Age (years)																			
18–64[1]	No	30486	94.9%	1645	5.1%	1	-	31794	99.0%	337	1.0%	-		32093	99.9%	38	0.1%	-	
	Yes	902	97.2%	26	2.8%	0.54 (0.35-0.78)	46.0% (21.7–64.8)	925	99.7%	3	0.3%	-		926	99.8%	2	0.2%	-	
65–79[2]	No	5064	79.4%	1316	20.6%	1	-	6062	95.0%	318	5.0%	-		6236	97.7%	144	2.3%	-	
	Yes	199	88.8%	25	11.2%	0.47 (0.30–0.70)	53% (30.3–70.3)	222	99.1%	2	0.9%	-		220	98.2%	4	1.8%	-	
≥80[2]	No	2380	67.6%	1142	32.4%	1	-	3500	99.4%	22	0.6%	-		3191	90.6%	331	9.4%	1	-
	Yes	510	83.6%	100	16.4%	0.40 (0.32–0.51)	60.0% (49.4–68.3)	610	100%	0	0.0%	-		565	92.6%	45	7.4%	0.76 (0.54–1.05)	*n*.R.

* only hospital-based mortalities were included in the analysis

(-): not calculated. Analyses were performed for age categories and outcomes with at least five individuals in each category. N.R.: not reported.

[1] Odds Ratio adjusted for sex, age and time period between SARS-CoV-2 test and the start of study (in weeks).

[2] Odds Ratio adjusted for sex and time period between SARS-CoV-2 test and the start of study (in weeks)
